# Comparison between Topical and Oral Tranexamic Acid in Management of Traumatic Hyphema

**Published:** 2014-03

**Authors:** Seyed Hamid Reza Jahadi Hosseini, Mohammad Reza Khalili, Mahmoud Motallebi

**Affiliations:** Poostchi Eye Research Center, Poostchi Clinic, Shiraz University of Medical Sciences, Shiraz, Iran;Department of Ophthalmology, Khalili Hospital, Shiraz University of Medical Sciences, Shiraz, Iran

**Keywords:** Hyphema, Topical, Tranexamic acid, Management

## Abstract

**Background: **We sought to determine the efficacy of topical tranexamic acid (5%) in the management of traumatic hyphema.

**Methods:** Thirty eyes with gross traumatic hyphema were enrolled in this study. The patients were treated with tranexamic acid (5%) eye drop every 6 hours for 5 days. The main outcome measures were best corrected visual acuity (BCVA), Intra-ocular pressure (IOP), day of clot absorption, and rate of rebleeding. These parameters were evaluated daily for 4 days and thereafter at the 8^th^ and 14^th^ days after treatment. The patients were also compared with two historical control groups of patients (80 eyes) with traumatic hyphema; the first control group was treated with oral placebo and the other group was treated with oral tranexamic acid at our department.

**Result: **Prior to treatment, the mean logarithm of the minimum angle of resolution (logMAR) BCVA was 0.59±0.62. BCVA was increased to 0.08±0.14 at day 14 (P<0.001) and the mean IOP before treatment was 13.7±3.9 mm Hg, which was reduced to 11.4±1.8 mm Hg at day 14 (P=0.004). Rebleeding occurred in one (3.3%) patient on the 4^th^ day post treatment. Comparison between the case group and the other two historical control groups with respect to the rebleeding rate demonstrated statistically significant differences between the case group and the first control group (P=0.008) but no statistically significant differences between the case group and the second control group (P=0.25).

**Conclusion: **Topical tranexamic acid seems promising in the management of traumatic hyphema. However, the small sample size of the present study precludes the conclusion that topical tranexamic acid can replace the oral tranexamic acid.

## Introduction

We aimed to determine the safety and effectiveness of topical tranexamic acid (5%) in the management of patients with traumatic hyphema. To our knowledge, the present study is the first to evaluate the effectiveness of topical tranexamic acid in the management of patients with hyphema.

Hyphema is defined as bleeding inside the anterior chamber of the eye. It has different etiologies, including trauma, coagulation disorders, herpetic disease, juvenile xanthogranuloma, retinoblastoma, leukemia, and rubeosis iridis. The most common etiology is ocular trauma, and hyphema occurs usually in cases of closed as well as open globe injuries.^1^^,^^2^ Traumatic hyphema, regardless of its size, often occurs in young men secondary to injury to the vessels of the peripheral iris or the anterior ciliary body. One of the most important complications of hyphema is rebleeding (secondary hemorrhage), which occurs in 3.5% to 38% (most studies report an incidence of less than 5%) of patients between 2 and 5 days after injury.^3^ Complications associated with secondary hemorrhage include glaucoma, optic atrophy, corneal blood staining, amblyopia, and posterior/anterior synechia. These complications may lead to surgical intervention.^4^ Thus, the prevention of rebleeding is important in the management of traumatic hyphema. Rebleeding results from the lysis and retraction of the fibrin clot that has occluded the bleeder vessel.^5^^,^^6^ Previous studies have shown that systemic antifibrinolytic agents reduce the incidence of rebleeding after traumatic hyphema.^7^ Therefore, antifibrinolytic agents such as E-aminocaproic acid and tranexamic acid prevent the activation of plasminogen.^8^^-^^10^ Tranexamic acid is 8 to 10 times more potent than aminocaproic acid. Tranexamic acid prevents clot lysis by occupying the lysine-binding site on plasminogen, activated plasmin, and prevents plasminogen and plasmin from binding to fibrin, which is necessary for clot lysis.^11^ Antifibrinolytic agents are contraindicated in the presence of active intravascular clotting such as diffuse intravascular coagulation (DIC) as well as in patients with pregnancy, coagulopathies, renal disease, platelet inhibition therapy, and hepatic disease. There are possible adverse reactions, including nausea, vomiting, muscle cramps, conjunctival suffusion, nasal congestion, headache, rash, pruritus, dyspnea, tonic toxic confusional states, cardiac arrhythmias, systemic hypotension, and gastrointestinal side effects.^2^^,^^3^

In our previous study,^12^ we showed that the topical administration of tranexamic acid was effective in yielding therapeutic intraocular concentrations of the drug without any ocular or systemic toxicity. After the administration of a single drop of 5% solution of tranexamic acid, aqueous concentrations of tranexamic acid reached >1.5 µg/ml within 160 minutes and then decreased to the average concentration of 1 µg/ml in 300 minutes, and it was detectable for up to 9 hours after administration.

## Patients and Methods

This is a comparative study conducted on 30 eyes with traumatic gross hyphema. For all the patients, complete general and ophthalmic examination was performed by an expert ophthalmologist before enrollment. Patients diagnosed with microscopic hyphema, ruptured globe, or posterior segment injuries other than commotio retina on the initial emergency department visit, those with any systemic disorders such as diabetes mellitus, hypertension, and coagulative disorders, those who used any anticoagulative medication or had a past history of ocular surgery, children under 7 years old, and pregnant and nursing women were excluded from the study. Best corrected visual acuity (BCVA) was measured using the Snellen chart.^13^ Relative afferent pupillary defect was checked. Slit lamp (HAAG-STREIT, Swiss made) examination was performed, and the percentage and location of layer hyphema was recorded. Intra-ocular pressure (IOP) was measured using the Goldmann Applanation Tonometer (HAAG-STREIT, BM900, Swiss made). Hyphema measurements were graded between 1 and 4 according to table 1.^14^

**Table 1:** Hyphema grading

**Grade**	**Hyphema**
1	If the layer of blood occupies less than one third of the anterior chamber
2	If the layer of blood fills one third to one half of the anterior chamber
3	If the layer of blood fills one half to less than the total volume of the anterior chamber
4	If there is total clotted hyphema (black ball or eight-ball hyphema)

In this case study, grades 3 and 4 were considered as one group due to the low number of the patients. Fundus examination (Keeler funduscope, U.K. made) was performed if visible. For all the patients, bed rest activity, semi-sitting position, and eye shield protection were recommended. The patients were treated with tranexamic acid (5%) eye drop [one ampoule tranexamic acid (500 mg/5ml) (TRANEXIP), Caspian Tamin Pharmaceutical Co., in 5 ml of artificial tear eye drop (TEARLOSE) containing hydroxypropyl methyl, cellulose, and dextran (Sina Daru Pharmaceutical Co.)] for 5 days every 6 hours. If corneal epithelium abrasion was observed, chloramphenicol eye drop was added every 6 hours. If IOP was >22 mm Hg, one or two topical anti-glaucoma medications were added. The main outcome measures were BCVA, IOP, day of clot absorption, and rate of rebleeding. These parameters were evaluated daily for 4 days and thereafter at the 8^th^ and 14^th^ days after the treatment began. The BCVA and IOP values were compared with these parameters before the treatment. In each follow-up visit, the patients were asked about subjective changes or side effects and they were checked for any objective ocular or systemic side effect of tranexamic acid. The patients in the present study were compared (chi-squared test and *t* test) with two historical control groups of patients with traumatic hyphema who had previously been treated; one group with oral placebo and the other one with oral tranexamic acid at our department.^10^ Both studies were done after our institutional Ethics Committee had approved the study protocol and informed consent had been obtained from all the participants (Ethics Committee’s code number: 89-01-19-2016). All the statistical analyses were performed using Statistical Package for Social Sciences software, version 16 (SPSS Inc., Chicago, IL, USA). A P<0.05 was considered statistically significant.

## Results

Thirty eyes of 30 patients at a mean age of 27.4±10.6 years old, ranging from 8 to 48, were included in this study. Twenty four (80%) patients were male and 6 (20%) were female. Eighteen (60%) eyes were right eyes and 12 (40%) were left eyes. Twenty-two (73.3%) patients had grade 1, 5 (16.7%) had grade 2, and 3 (10%) had grade 3 layer hyphema. No patient had grade 4 hyphema. The mean logarithm of the minimum angle of resolution (logMAR) BCVA before treatment was 0.59±0.62, with a range of 0.00 to 3.00, which changed to 0.08±0.14, ranging from 0.00 to 0.70, on day 14 (P<0.001). The mean IOP before treatment was 13.7±3.9 mm Hg, ranging from 8 to 28 mm Hg, which decreased to 11.4±1.8 mm Hg, ranging from 9 to 16 mm Hg on day 14 (P=0.004). The mean day of clot absorption was 4.1±1.7 days. Rebleeding occurred only in one (3.3%) patient on day 4. This patient had grade 2 layer hyphema initially and topical tranexamic acid was started 8 hours after trauma. Because of rebleeding, conventional treatment (oral tranexamic acid) was started and final logMAR BCVA was 0.1 and IOP was 12 mm Hg. The topically applied tranexamic acid was well tolerated locally, and no patient experienced ocular and systemic side effects. In one patient, BCVA on day 14 was reduced compared with baseline; evaluation of the macula by optical coherence tomography (OCT) showed a macular hole and the patient was referred to the Posterior Segment Clinic.

Comparison was made between the patients in this study and two historical control groups. The first historical control group, which was treated with oral placebo in this department, comprised 80 patients [66 (82%) males and 18 (18%) females] with hyphema at a mean age of 14.8±10.7 years old (range=3-58 years old) with the same race and demographic characteristics . Twenty-one (26%) patients in this group experienced rebleeding; therefore, there were statistically significant differences between the case group and this control group in terms of the rebleeding rate (P=0.008). The second historical control group, which was treated with oral  tranexamic acid in this department, consisted of 80 patients [63 (79%) males and 17 (21%) females] with hyphema at a mean age of 14.9±12.6 years old (range=1 to 65 years old) with the same race and demographic characteristics. Eight (10%) patients in this group experienced rebleeding; as a result, there were no statistically significant differences between the case group and this historical control group as regards the rebleeding rate (P=0.25) (tables 2 to 5).

**Table 2:** Sex, laterality, hyphema level, and rebleeding in the oral placebo and topical tranexamic acid groups

**Variable**	**Oral placebo** **(historical control group 1)**	**Topical tranexamic acid** **(case group)**	**Chi-square (p)**
	**(n=80) [no.(%)]**	**(n=30) [no.(%)]**	
Sex (male)	66 (82)	24 (80)	0.81
Eye (right)	39 (49)	18 (60)	0.307
Grade 1 hyphema	63 (79)	22 (73.3)	0.528
Grade 2 hyphema	13 (16)	5 (16.7)	0.936
Grades 3 and 4 hyphema	4 (5)	3 (10)	0.342
Rebleeding	21 (26)	1 (3.3)	0.008

**Table 3:** Mean age, IOP,* hyphema, clearance, and day of rebleeding in the oral placebo and topical tranexamic acid groups

**Variable**	**Oral placebo** **(historical control group 1)**		**Topical tranexamic acid** **(case group)**		***t *** **test (p)**
	**(n=80)**		**(n=30)**		
	**mean (SD)**	**range**	**mean (SD)**	**range**	
Age (y/o)	14.8 (10.7)	3-58	27.4 (10.6)	8-48	0.001
IOP (mm Hg) before treatment	18 (9.2)	3-48	13.7 (3.9)	8-28	0.001
IOP (mm Hg) after treatment	12.1 (6.8)	3-26	11.4 (18)	9-16	0.05
Hyphema clearance (day)	3.7 (1.6)	1-8	4.1 (1.7)	2-8	0.20
Day of rebleeding	3.8 (1.0)	2-6	4	4	0.001

*Intra-ocular pressure

**Table 4:** Sex, laterality, hyphema level, and rebleeding in the oral and topical tranexamic acid groups

**Variable**	**Systemic tranexamic acid (historical control group 2)**	**Topical tranexamic acid (case group)**	**Chi-square (p)**
	**(n=80) [no.(%)]**	**(n=30) [no.(%)]**	
Sex (male)	63 (79)	24 (80)	0.912
Eye (right)	39 (49)	18 (60)	0.003
Grade 1 hyphema	62 (77.5)	22 (73.3)	0.067
Grade 2 hyphema	13 (16.25)	5 (16.7)	0.960
Grades 3 and 4 hyphema	5 (6.25)	3 (10)	0.502
Rebleeding	8 (10)	1 (3.3)	0.254

**Table 5:** Mean age, IOP,* hyphema clearance, and day of rebleeding in the oral and topical tranexamic acid groups

**Variable**	**Systemic tranexamic acid (historical control group 2)**		**Topical tranexamic acid (case group)**		***t*** ** test (p)**
	**(n=80)**		**(n=30)**		
	**mean (SD)**	**range**	**mean (SD)**	**range**	
Age (y/o)	14.9 (12.6)	1-65	27.4 (10.6)	8-48	0.001
IOP (mm Hg) before treatment	17.8 (6)	9-36	13.7 (3.9)	8-28	0.001
IOP (mm Hg) after treatment	10.5 (4.3)	9-17	11.4 (18)	9-16	0.013
Hyphema clearance (day)	4 (2.2)	1-11	4.1 (1.7)	2-8	0.07
Day of rebleeding	3.4 (0.7)	2-4	4	4	0.001

*Intra-ocular pressure

## Discussion

This study may provide evidence that topical tranexamic acid is safe and could be an effective alternative to oral treatment to reduce the incidence of secondary hemorrhage in traumatic hyphema. According to the results, the mean day of clot absorption was 4.1±1.7 days and rebleeding occurred in only one (3.3%) patient on day 4. Comparison (power for the chi-squared test of 88.5%) of the rates of rebleeding between the patients in this study (1/30) and the first historical control group [comprising 80 patients with hyphema treated with oral placebo at our department (26/80)] demonstrated statistically significant differences. In contrast, comparison (power for the chi-squared test of 54.8%) of the rates of rebleeding between the case group and the second historical control group [comprising 80 patients with hyphema treated with oral tranexamic acid at our department (8/80)] demonstrated no statistically significant differences.^10^ Although topical tranexamic acid was shown to be effective in the management of traumatic hyphema, it cannot be a certain substitute for oral  tranexamic acid due to the small number of cases.

Oral administration is a major route of drug administration; nevertheless, the orally administered drugs must reach the intraocular tissue and fluids through the blood circulation. Moreover, due to blood–ocular barriers, large amounts of the drug and frequent administrations are required to maintain therapeutic concentrations, which may result in drug intolerance because of serious side effects. Local or organ-specific administration of the drug is desirable because of the potential to reduce or eliminate systemic toxicities and to improve therapeutic efficacy. The eye is one of the most ideal sites in the human body for direct drug delivery because the intraocular structures are relatively easy to access. Be that as it may, they are isolated from the systemic circulation by blood–ocular barriers. These barriers minimize systemic absorption and side effects.^15^ To justify the topical administration of tranexamic acid, an important question is whether fibrinolysis occurs at the aqueous or vascular side of the clot. Topical tranexamic acid may be an attractive alternative to systemic delivery in the treatment of traumatic hyphema, but the efficacy of topical treatment has been questioned. The answer to this question determines whether tranexamic acid should reach the vascular or the intraocular side. Tissue plasminogen activator and urokinase-type plasminogen activator are present in the aqueous humor normally and an intensive plasminogenesis exists in the aqueous humor. The activity of plasminogen activator inhibitors in the aqueous humor is negligible. A high concentration of fibrin degradation products exists in the aqueous of patients with rebleeding after traumatic hyphema.^16^^,^^17^ Furthermore, another important antifibrinolytic agent, aminocaproic acid, when applied topically in animal and human models, has been effective in the prevention of rebleeding in traumatic hyphema.^18^ Based on such evidence, topical tranexamic acid might be effective in the prevention of rebleeding in patients with traumatic hyphema. Another question to be answered is whether the topical administration of tranexamic acid is effective in yielding therapeutic intraocular concentrations. Astedt^11^ reported that the therapeutic concentration of tranexamic acid in serum was 8-10 micgr/ml and aqueous concentration was 10% of the serum concentration. Therefore, 0.8-1 micgr/ml aqueous concentration of the drug was enough to prevent fibrinolysis in patients with hyphema. Bramsen^19^ showed that aqueous concentration, followed by a single dose of oral tranexamic acid (25 mg/kg), was 1.6 micgr/ml after 3 hours. In our previous study,^12^ we demonstrated that the aqueous concentration of the drug after the administration of a single drop of 5% tranexamic acid solution was higher than 1.5 micgr/ml up to 160 minutes, and 1 micgr/ml at 300 minutes remained nearly unchanged for up to 9 hours after administration. Thus, it seems that the topical administration of tranexamic acid is effective in providing adequate therapeutic intraocular concentrations.

The results of the present study clearly demonstrate the effectiveness of topical tranexamic acid in the prevention of rebleeding in patients with traumatic hyphema. Among our study population, the rebleeding rate was 3.3%, which is similar to the rate of rebleeding in the previous studies that used oral tranexamic acid, systemic aminocaproid acid, topical aminocaproic acid, and systemic corticosteroid to prevent rebleeding in patients with traumatic hyphema. (The rebleeding rate is 3% to 30% in these studies.)^1^^-^^10^ No ocular side effect was detected, and the topically applied tranexamic acid was well tolerated without evidence of systemic toxicity.

The small number of the cases and the differences in the mean ages between the two groups could be considered as the limitations of this study. Although some bias is present, we compared each patient to himself/herself before and after treatment. Further double-masked clinical trial studies with larger numbers of cases are required to confirm the finding of this study.

## Conclusion

This study provides evidence that topical tranexamic acid seems to be effective in the management of traumatic hyphema. However, our small sample size precludes the conclusion that topical tranexamic acid can replace oral tranexamic acid. 
